# Prognostic and predictive value of radiomic signature in stage I lung adenocarcinomas following complete lobectomy

**DOI:** 10.1186/s12967-022-03547-9

**Published:** 2022-07-28

**Authors:** Wei Nie, Guangyu Tao, Zhenghai Lu, Jie Qian, Yaqiong Ge, Shuyuan Wang, Xueyan Zhang, Hua Zhong, Hong Yu

**Affiliations:** 1 Department of Pulmonary Medicine, Shanghai Chest Hospital, School of Medicine, Shanghai Jiao Tong University, Shanghai, China; 2 Department of Radiology, Shanghai Chest Hospital, School of Medicine, Shanghai Jiao Tong University, Shanghai, China; 3 Department of Radiology, Xinhua Hospital, Shanghai Jiao Tong University School of Medicine, Shanghai, China; 4 Department of Emergency Medicine, Shanghai Chest Hospital, School of Medicine, Shanghai Jiao Tong University, Shanghai, China; 5 General Electric (GE) Healthcare, Shanghai, China

**Keywords:** Radiomic signature, Adjuvant chemotherapy, Lung adenocarcinoma, Overall survival

## Abstract

**Background:**

The overall survival (OS) of stage I operable lung cancer is relatively low, and not all patients can benefit from adjuvant chemotherapy. This study aimed to develop and validate a radiomic signature (RS) for prediction of OS and adjuvant chemotherapy candidates in stage I lung adenocarcinoma.

**Methods:**

A total of 474 patients from 2 centers were divided into 1 training (n = 287), 1 internal validation (n = 122), and 1 external validation (n = 65) cohorts. We extracted 1218 radiomic features from preoperative CT images and constructed RS. We further investigated the prognostic value of the RS in survival analysis. Interaction between treatment and RS was assessed to evaluate its predictive value. Propensity score matching (PSM) was conducted.

**Results:**

Overall, 474 eligible patients with stage I lung adenocarcinoma (214 men [45.1%]; median age, 60 years) were identified. The RS was significantly associated with OS in the training and two validation cohorts (hazard ratios [HRs]  >  = 3.22). In multivariable analysis, the RS remained an independent prognostic factor adjusting for clinicopathologic variables (adjusted HRs >  = 2.63). The prognostic value of RS was also confirmed in PSM analysis. In stage I patients, the interaction between RS status and adjuvant chemotherapy was significant (interaction *P* = 0.020). Within the stratified analysis, good chemotherapy efficacy was only observed for patients with stage IB disease (interaction *P* < 0.001).

**Conclusions:**

Our results suggested that the radiomic signature was associated with overall survival in patients with stage I lung adenocarcinoma and might predict adjuvant chemotherapy benefit, especially in stage IB patients. The potential of radiomic signature as a noninvasive predictor needed to be confirmed in future studies.

**Supplementary Information:**

The online version contains supplementary material available at 10.1186/s12967-022-03547-9.

## Background

Surgery with curative intent is the foundation for management of early-stage non-small-cell lung cancer (NSCLC). However, 10–20% of stage IA and 30% of stage IB patients will die within 5 years of surgery [1]. For stage IA patients, adjuvant chemotherapy is not recommended by American Society of Clinical Oncology (ASCO) and National Comprehensive Cancer Network (NCCN) guidelines [2, 3]. For stage IB patients, different guidelines give discordant recommendations [2, 3]. Therefore, it is important to find a clinically actionable biomarker for the prediction of prognosis and adjuvant chemotherapy benefits in patients with resected stage I NSCLC.

Computed tomography (CT) is routinely used in lung cancer diagnosis and could provide the possibility of calculating prognostic and predictive biomarkers for patients’ management. CT-based radiomics has become an attractive method for predicting gene mutations, treatment sensitivity, and prognosis in NSCLC [4–6]. Recently, Xie et al. indicated that the radiomics nomogram could be used for prognostic prediction (hazard ratio [HR] = 7.794; 95% confidence interval [CI]: 3.185–19.078; *P* < 0.001) and adjuvant chemotherapy benefits identification for patient with resected stage I lung adenocarcinoma (*P* = 0.040) [7]. However, only disease-free survival (DFS) was assessed in that study.

The goal of cancer treatment is to improve patient survival. Overall survival (OS) is an optimal end point which indicates patient benefit. In the present study, we developed and validated a radiomic signature (RS) to predict OS in lobectomized stage I lung adenocarcinoma, and then set out to explore if the RS could be a biomarker for identification patients who can benefit from adjuvant chemotherapy.

## Methods

### Study design and patient population

This was a retrospective multicenter study including three independent cohorts (n = 474, Additional file 1: Figure S1). The training cohort and internal validation cohort included 287 and 122 patients who were treated in Shanghai Chest Hospital from March 2009 to December 2013 and January 2014 to May 2016, respectively. The external validation cohort included 65 patients who were treated in Xinhua Hospital from February 2015 to March 2021.

All patients diagnosed with stage I lung adenocarcinoma according to the 8th TNM edition of the American Joint Committee on Cancer (AJCC) cancer staging manual were identified [8]. Patients were excluded based on the following criteria: (1) multiple primary lung adenocarcinoma; (2) adenocarcinoma in situ and microinvasive adenocarcinoma (MIA); (3) without high resolution CT images carried out less than 2 weeks before surgery; (4) died within 1 month after surgery; (5) lost to follow-up.

The demographic and clinicopathological characteristics were collected from medical records databases in two hospitals. Platinum-based doublet chemotherapy was used as the basic regimen. The platinum drugs included cisplatin and carboplatin, and other drugs included vinorelbine, paclitaxel, gemcitabine or pemetrexed.

Ethics committee approval was obtained from the institutional review boards at Shanghai Chest Hospital (KS1596, 08/28/2019) and Xinhua Hospital (XHEC-D-2021–137, 10/18/2021). Informed consent was waived because data were deidentified.

### Outcome and follow-up

The primary endpoint was OS, which was defined as the time from surgery to death from any cause. All patients were postoperatively followed every 3 months during the first 2 years and then every 6 months annually thereafter. The clinical evaluations included physical examination, blood tests, chest CT, abdominal CT, or ultrasound. Whole-body bone scans and a cranial CT or magnetic resonance imaging were performed annually.

### CT data acquisition and imaging feature detection

Chest CT scans were performed using the following 4 scanners: Discovery CT750HD CT scanner (GE, Waukesha, WI, USA), 256-detector row scanner (Revolution CT, GE, Waukesha, WI, USA), 64-detector row scanner (Brilliance, Philips, Cleveland, OH, USA), and a 16-detector row scanner (uCT S160, United Imaging, Shanghai, China). Patients were scanned at the end of inspiration during a single breath hold in the supine position. The HRCTs were performed with collimation of 0.625–1.25 mm, pitch of 0.64, section thickness of 0.625–1.25 mm without overlap, matrix of 512 × 512 or 1,024 × 1,024, field of view (FOV) of 350–400 mm, 120 kVp, and 220–300 mA. All imaging data were reconstructed using the standard algorithm. One radiologist (G.T., with 10 years of experience in chest CT interpretation) identified manually at the voxel level the areas of interest for the included nodules based on CT scans using 3D Slicer (version 4.8.0, Brigham and Women’s Hospital, Boston, MA, USA). Then, the VOI was confirmed by another radiologist (H.Y., with 30 years of experience in chest CT interpretation).

Radiomics features were extracted by Image Biomarker Standardization Initiative (IBSI) compliant AK software (Analysis Kit Software, Version3.3.0, GE Healthcare). Totally, 1218 radiomic features were extracted from CT images, including first order statistical features, morphological features, gray-level co-occurrence features matrix-based features, gray-level run length matrix-based features, gray-level size zone matrix-based features, gray-level dependence matrix-based features, and the transform features of wavelet and Laplace changes.

### Radiomic signature construction and validation

The RS was calculated with chest CT based on the training cohort. Univariate Cox analysis was firstly used to detect the associations between each feature and the patients’ OS. The features with *P* < 0.05 were used for further analysis. The Spearman correlation was applied to eliminate the redundancy of the feature set (coefficient of chosen here | r |> 0.8). Finally, the least absolute shrinkage and selection operator (LASSO) method to select the most valuable prognostic features from the training cohort. The optimal cutoff value for RS was determined using X-tile software version 3.6.1 (Yale University School of Medicine, New Haven, CT, USA) in the training cohort [9]. The same cutoff value was applied to all the validation cohorts. The patients were divided into high and low risk groups in each cohort. The time dependent receiver operating characteristic (ROC) curve was created to assess the prognostic accuracy of the RS in the training and two validation cohorts. To adjust for selection bias, propensity score matching (PSM) was did, due to the imbalanced data between low and high risk groups. The propensity score was assessed for training and internal validation sets [10]. Nearest neighbor matching was selected and matched controls were not replaced during matching. Using the propensity scores, high risk patients were randomly matched to low risk patients with 1:1 matching method.

### Statistical analysis

Mann–Whitney *U* test was used to examine the difference between the two groups. Categorical data were compared using the χ2-test or Fisher’s exact test, as appropriate. OS was calculated using the Kaplan–Meier method and log-rank test. The univariate and multivariate Cox proportional hazards model was utilized to estimate the HR and 95% CI for the outcome. Interaction between the RS and adjuvant chemotherapy was assessed by means of the Cox model.

We established a clinicopathologic model and a radiomic nomogram to determine whether the RS added incremental value for predicting OS. Model performance was assessed by Harrell’s concordance index (C-index), calibration curves and decision curve analysis.

For all analyses, *P* < 0.05 was considered statistically significant in all 2-tailed tests. The statistical analyses were performed using R version 3.6.1 (R Project for Statistical Computing) and SPSS version 23.0 (IBM, Armonk, NY).

## Results

### The characteristics of the patient population

Table 1 listed the detailed demographic and clinicopathological characteristics of the patients in the training (n = 287), internal validation (n = 122), and external validation (n = 65) cohorts. The median age of the entire cohort was 60 years and 214 (45.1%) were man. The median follow-up time for OS were 92.3 months in the training cohort, 92.4 months in the internal validation cohort, and 26.6 months in the external validation cohort.

**Table 1:** Demographic and clinicopathological characteristics

	Training cohort (n = 287)	Internal validation cohort (n = 122)	External validation cohort (n = 65)
Age, median (IQR)	60 (54–67)	60 (51–66.3)	61 (52–69)
Gender (%)			
Male	127 (44.3)	60 (49.2)	27 (41.5)
Female	160 (55.7)	62 (50.8)	38 (58.5)
Smoking history (%)			
Never	254 (88.5)	110 (90.2)	40 (61.5)
Ever	33 (11.5)	12 (9.8)	25 (38.5)
Tumor location (%)			
Upper left	58 (20.2)	23 (18.9)	15 (23.1)
Lower left	40 (13.9)	20 (16.4)	11 (16.9)
Upper right	102 (35.5)	47 (38.5)	19 (29.2)
Right middle	32 (11.1)	5 (4.1)	8 (12.3)
Lower right	55 (19.2)	27 (22.1)	12 (18.5)
Pathological subtype (%)			
Lepidic	36 (12.5)	12 (9.8)	19 (29.2)
Acinar/papillary	236 (82.2)	106 (86.9)	40 (61.5)
Solid/micropapillary	15 (5.2)	4 (3.3)	6 (9.2)
Tumor size (cm)			
0–1	47 (16.4)	23 (18.9)	22 (33.8)
1–2	146 (50.9)	53 (43.4)	17 (26.2)
2–3	73 (25.3)	36 (29.5)	18 (27.7)
3–4	21 (7.3)	10 (8.2)	8 (12.3)
TNM stage (%)			
IA1	45 (15.7)	20 (16.4)	22 (33.8)
IA2	130 (45.3)	45 (36.9)	10 (15.4)
IA3	62 (21.6)	31 (25.4)	7 (10.8)
IB	50 (17.4)	26 (21.3)	26 (40.0)
Visceral pleural invasion (VPI, %)			
Absent	247 (86.1)	101 (82.8)	45 (69.2)
Present	40 (13.9)	21 (17.2)	20 (30.8)
Lymphovascular invasion (LVI, %)			
Absent	278 (96.9)	119 (97.5)	64 (98.5)
Present	9 (3.1)	3 (2.5)	1 (1.5)
Duration of follow-up, median (month)	92.3	92.4	26.6
Adjuvant chemotherapy (%)	32 (11.1)	11 (9.0)	NA

*IQR* interquartile range, *NA* not available

### Radiomic signature construction and validation

**Fig. 1:**
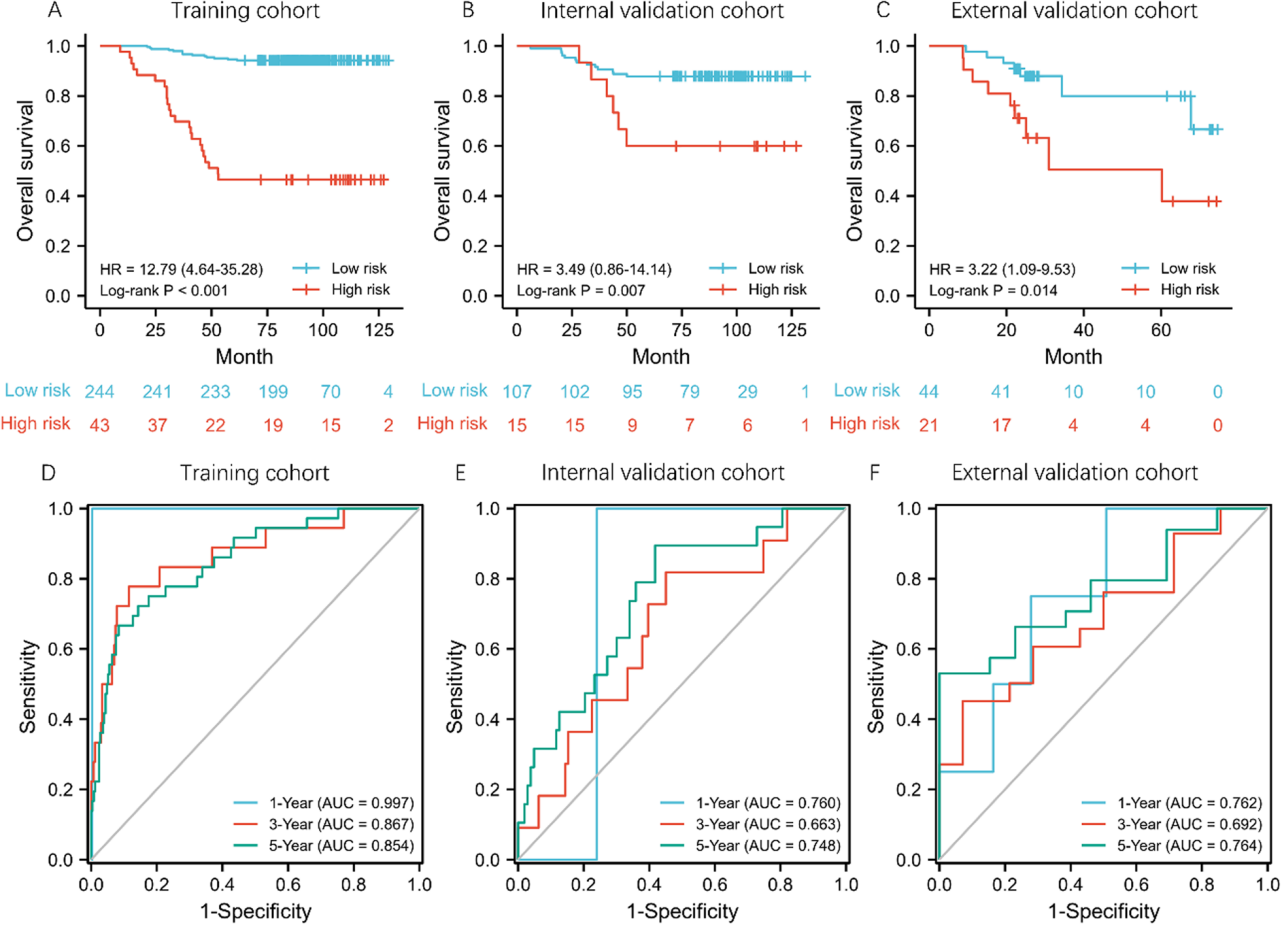
Kaplan–Meier and time-dependent receiver operating characteristic curves according to the radiomic signature. Kaplan–Meier estimates of overall survival in low and high risk groups in training cohort (**A**), internal validation cohort (**B**), and external validation cohort (**C**). P values were calculated using two-sided log-rank test. Area under the curves at 1 year, 3 years, and 5 years were calculated to assess the prognostic accuracy within the training cohort (**D**), internal validation cohort (**E**), and external validation cohort (F)

The calculation formula for RS was 0.456 * log_sigma_5_0_mm_3D_firstorder_Maximum + 0.355 * original_firstorder_Minimum + 0.236 * log_sigma_2_0_mm_3D_gldm_Large Dependence High Gray Level Emphasis + 0.218 * original_gldm_Large Dependence High Gray Level Emphasis + 0.149 * log_sigma_4_0_mm_3D_glszm_Small Area Low Gray Level Emphasis + 0.015 * wavelet_HLH_firstorder_Mean—0.072 * log_sigma_5_0_mm_3D_firstorder_Skewness. To determine the optimal cutoff value of RS, X-tile was used. The optimal cutoff value was 0.98 (approximately equal to 1) which showed the most significant prognostic effect in predicting OS in the training cohort. Therefore, RS of 1 was used as the cutoff point in the following analyses. The Kaplan–Meier survival curves confirmed a significant difference in OS (HRs >  = 3.22) between the high and low risk groups (Fig. 1A–C). The results remained significant in multivariate analyses, after adjusting for clinical and pathologic factors (adjusted HRs >  = 2.63; Additional file 1: Table S1). The areas under the curve at different follow-up times (1, 3, and 5 years) also confirmed that the RS had good prognostic accuracy in the training and validation cohorts (Fig. 1D–F). Subgroup analyses suggested that the RS could also predict prognosis according to TNM stage and tumor size in each cohort (Additional file 1: Figure S2).

The characteristics for the low and high risk groups of the training and validation combination cohorts are shown in Additional file 1: Table S2. Significant differences were observed between the two groups. We thus did a PSM analysis. After matching, baseline characteristics were generally comparable between low and high risk patients (Additional file 1: Table S2). We also found that high risk patients had significantly shorter OS (Additional file 1: Figure S3).

Collectively, these results revealed that stage I lung adenocarcinoma patients with high risk had worse OS compared to those with low risk.

### Increasing prognostic value of the radiomic signature

**Fig. 2:**
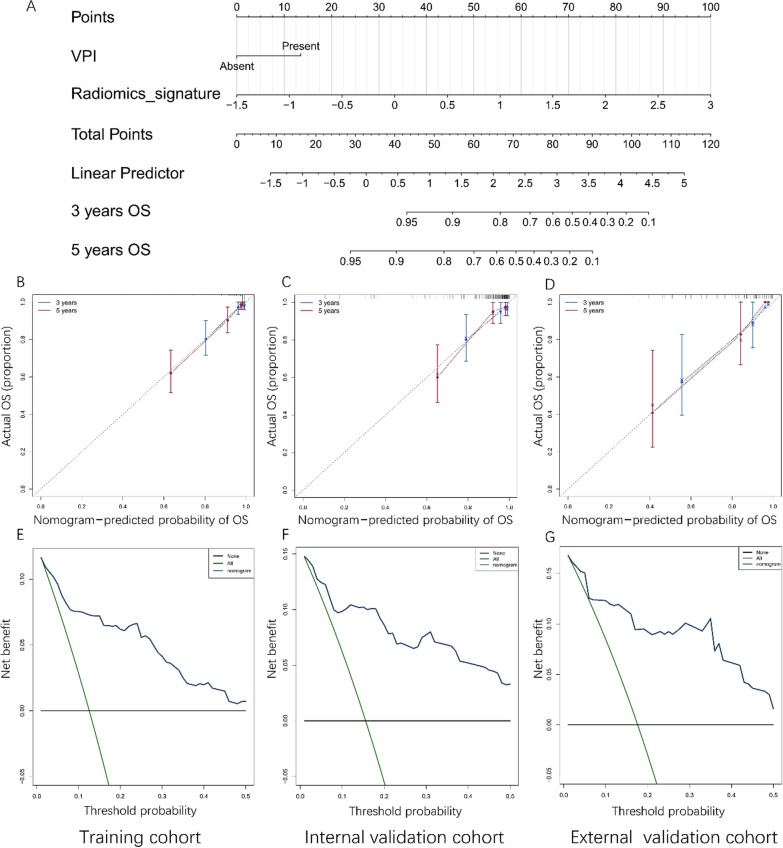
Nomogram, calibration curves, and decision curves to estimate overall survival. The radiomic nomogram for estimating overall survival (**A**). The calibration curves for the radiomic nomogram in the training and validation cohorts (**B**: the training cohort with n = 287; **C**: internal validation cohort with n = 122; **D**: external validation cohort with n = 65). The error bars were defined as s.e.m., which represent the 95% CI. The decision curves for the nomogram in the training and validation cohorts (**E**: the training cohort with n = 287; **F**: internal validation cohort with n = 122; G: external validation cohort with n = 65)

Multivariate Cox regression analysis suggested that visceral pleural invasion and RS could predict OS in the training cohort (Additional file 1: Table S1). Thus, a radiomic nomogram, based on the RS and the clinicopathologic factor, was constructed by using the training cohort (Fig. 2A). The calibration curves showed good agreement at 3 years and 5 years between prediction and observation in the probability of OS in the training and two validation cohorts (Fig. 2B–D). The C-index values revealed that the radiomic nomogram had better prognostic performance than the clinical nomogram (Additional file 1: Table S3). The decision curve analysis indicated that the radiomic nomogram had good clinical performance, with advantages across almost the entire range of reasonable threshold probabilities in the training and two validation cohorts (Fig. 2E–G). Consequently, these results revealed that the RS could provide additional value for OS prediction.

### Adjuvant chemotherapy benefit analysis based on the radiomic signature

**Fig. 3:**
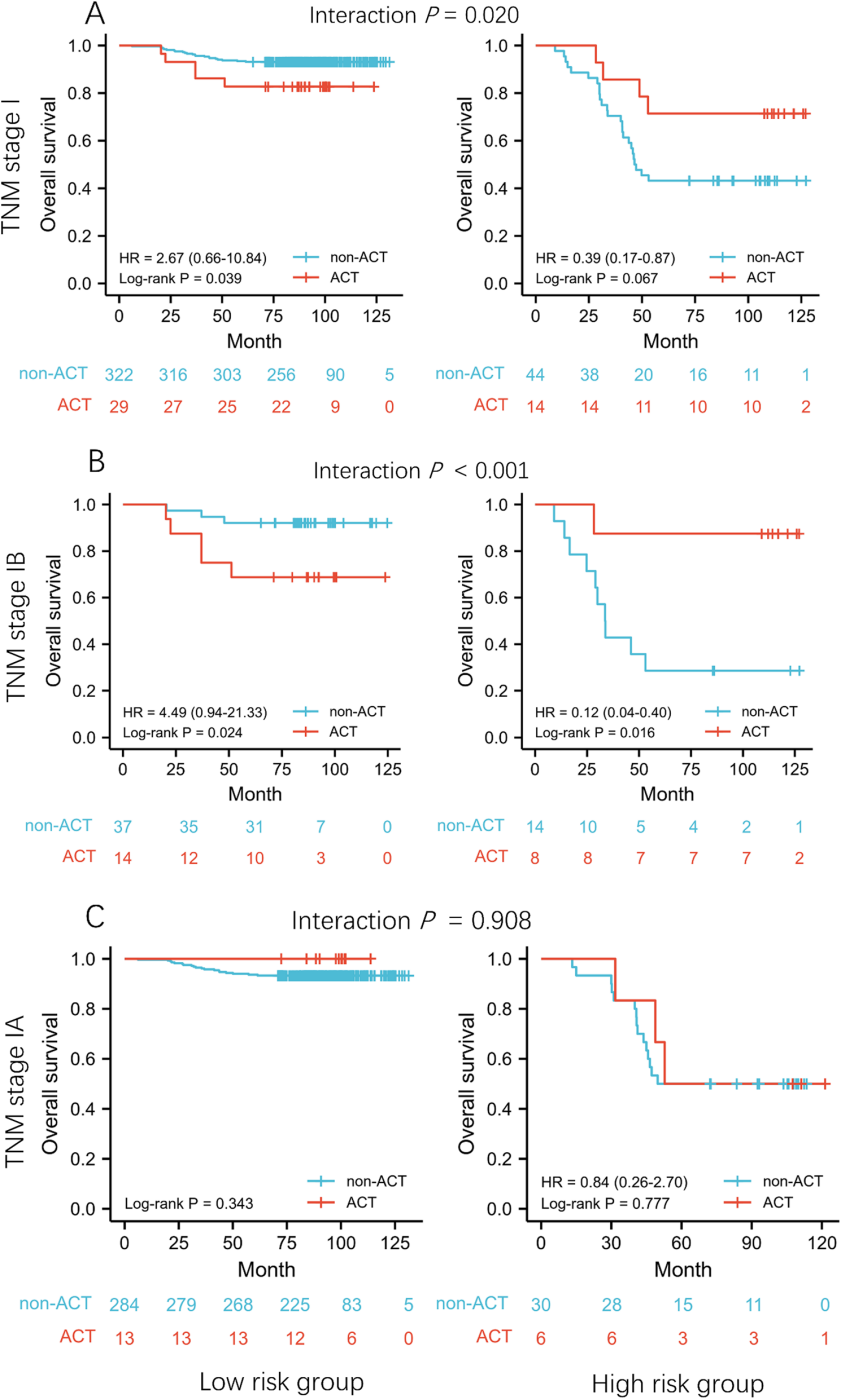
Kaplan–Meier curves according to treatment. Predictive capacity for OS is stratified by treatment with ACT vs. non-ACT in patients with low or high risk in stage I (**A**), stage IB (**B**), and stage IA (**C**). ACT, adjuvant chemotherapy

To assess the predictive value of RS, we evaluated the association between RS and OS among stage I patients from training and internal validation cohorts (n = 409) who either received or did not receive adjuvant chemotherapy. In stage I patients, the interaction between RS status and adjuvant chemotherapy was significant (interaction *P* = 0.020; Fig. 3A), suggesting the predictive role of RS for chemotherapy benefit. Then we did interaction tests for RS and adjuvant chemotherapy efficacy in stage IA and IB subgroups. The results revealed that high risk patients with stage IB could obtain OS benefit from the adjuvant chemotherapy (interaction *P* < 0.001; Fig. 3B), while no significant interaction was observed in the stage IA subgroup (interaction *P* = 0.908; Fig. 3C).

## Discussion

In the present study, we developed the RS by preoperative CT images and validated its ability to predict OS in three cohorts from two centers. More importantly, the RS might predict response to adjuvant chemotherapy in lobectomized stage I lung adenocarcinoma, especially in stage IB.

In a previous study, Xie and colleagues found that RS could predict RFS and identify the patients benefit from adjuvant chemotherapy in stage I adenocarcinoma patients (*P* = 0.040) [7]. Most studies about postoperative adjuvant chemotherapy used OS as the primary endpoint [11–13]. In an individual participant data meta-analysis, Burdett et al. determined a benefit of adding chemotherapy after surgery (HR = 0.86; 95% CI: 0.81–0.92; *P* < 0.001), with an absolute increase in OS of 4% at five years [12]. In addition, after analyzing 577 NSCLC patients from two data sets, Le et al. indicated that the risk score generated using CT-based radiomics signatures could predict overall survival in NSCLC patients [14]. Thus, we focused on OS in this study and also revealed RS's prognostic and predictive potential. We found that high RS was associated with solid/micropapillary subgroup and tumor size, which was consistent with the previous report [7]. When PSM was used to minimize potential selection bias and confounding effects, RS could also predict OS. Therefore, pathological subtypes and tumor size may not be the explanations for the relationship between RS and OS. Perez-Johnston et al. suggested that other clinicopathological and genomic features, such as tumor spread through air spaces, phosphoinositide 3-kinase pathway or *STK11* alterations, were enriched in certain CT-based radiomic clusters [15]. Therefore, it was possible that these clinicopathological and genomic features might influence the association between RS and OS.

In patients with stage IA, high RS was associated with worse OS. However, adjuvant chemotherapy could not improve survival in these high risk patients. Therefore, clinicians should formulate a detailed follow-up plan in order to detect local or metastatic relapse. Molecular residual disease (MRD) detection could precisely predict the recurrence in patients with NSCLC after definitive surgery [16]. It will be interesting to use MRD detection to predict survival in high risk patients with stage IA.

Poorly differentiated, lymphovascular invasion, visceral pleural invasion, incomplete lymph node sampling, or wedge resection were defined as high risk factors in stage IB by the current NCCN guideline [3]. Several studies reported stage IB patients with these high risk factors derived survival benefit from adjuvant chemotherapy [17, 18]. However, it is still controversial to identify patients with early-stage NSCLC who may benefit from adjuvant chemotherapy after surgery. In our study, improved OS was observed in adjuvant chemotherapy receivers with a high RS, suggesting its ability to predict survival and response to chemotherapy. However, the sample size of patients receiving adjuvant chemotherapy was moderate, which might limit the statistical power of conclusions. Thus, the radiomic signature as a noninvasive method should be assessed in the large-scale prospective studies.

Osimertinib has been recommended to use in patients with stage IB *EGFR* mutation-positive NSCLC [19]. In addition, radiomic signature may predict the prognosis of metastatic NSCLC patients with receiving osimertinib therapy [20]. Therefore, the association between the radiomic signature and efficacy of osimertinib as adjuvant therapy is needed to be investigated.

There were some limitations in our study. First, as a retrospective study, potential selection bias may hamper the reproducibility and comparability of the results. Thus, we included three independent cohorts from two medical centers to validate our findings. Second, genetic data was not included because gene detection was not a routine practice. Third, the role of radiomic signature was only assessed in Chinese patients. The performance of RS in other ethnic patients was still unknown. Finally, it would be interesting to perform experiments with cell lines and animal models to reveal the underlying mechanism of radiomic signature.

## Conclusions

In summary, our results showed that radiomic signature might be a promising biomarker to predict OS and benefit of adjuvant chemotherapy in resected stage I lung adenocarcinoma. Further prospective studies are warranted to validate these results.

## Supplementary Information

**Additional file 1: Figure S1.** The process of patient selection in the training and two validation cohorts. **Figure S2.** Kaplan-Meier overall survival curves according to the radiomic signature among stage I lung adenocarcinoma patient subgroups. The training cohort (A, D, G, J), The internal validation cohort (B, E, H, K), and external validation cohort (C, F, I, L). P values were calculated using two-sided log-rank test. **Figure S3.** Kaplan-Meier overall survival curve according to the radiomic signature after propensity score matching. **Table S1.** Multivariate Cox Regression analyses for overall survival in the training and validation cohorts. **Table S2.** Demographic and clinicopathological characteristics of patients by radiomic signature level before and after propensity score matching. **Table S3.** The performances of the different models in the training and validation cohorts.

## Data Availability

Any reasonable requests for access to available data underlying the results reported in this article will be considered. Such proposals should be submitted to the corresponding author.

## Abbreviations

OS: Overall survival
RS: Radiomic signature
PSM: Propensity score matching
HR: Hazard ratio
NSCLC: Non-small-cell lung cancer
ASCO: American society of clinical oncology
NCCN: National comprehensive cancer network
CT: Computed tomography
DFS: Disease-free survival
AJCC: American joint committee on cancer
MIA: Microinvasive adenocarcinoma
LASSO: Least absolute shrinkage and selection operator
ROC: Receiver operating characteristic
