# Supplementary material for: Prognostic and predictive value of radiomic signature in stage I lung adenocarcinomas following complete lobectomy
Source: J Transl Med. 2022 Jul 28;20:339. doi: 10.1186/s12967-022-03547-9 (PMC9331779; doi:10.1186/s12967-022-03547-9)
Supplement: Supplementary file 1 — Additional file 1: Figure S1. The process of patient selection in the training and two validation cohorts. Figure S2. Kaplan-Meier overall survival curves according to the radiomic signature among stage I lung adenocarcinoma patient subgroups. The training cohort (A, D, G, J), The internal validation cohort (B, E, H, K), and external validation cohort (C, F, I, L). P values were calculated using two-sided log-rank test. Figure S3. Kaplan-Meier overall survival curve according to the radiomic signature after propensity score matching. Table S1. Multivariate Cox Regression analyses for overall survival in the training and validation cohorts. Table S2. Demographic and clinicopathological characteristics of patients by radiomic signature level before and after propensity score matching. Table S3. The performances of the different models in the training and validation cohorts. [file 12967_2022_3547_MOESM1_ESM.docx]

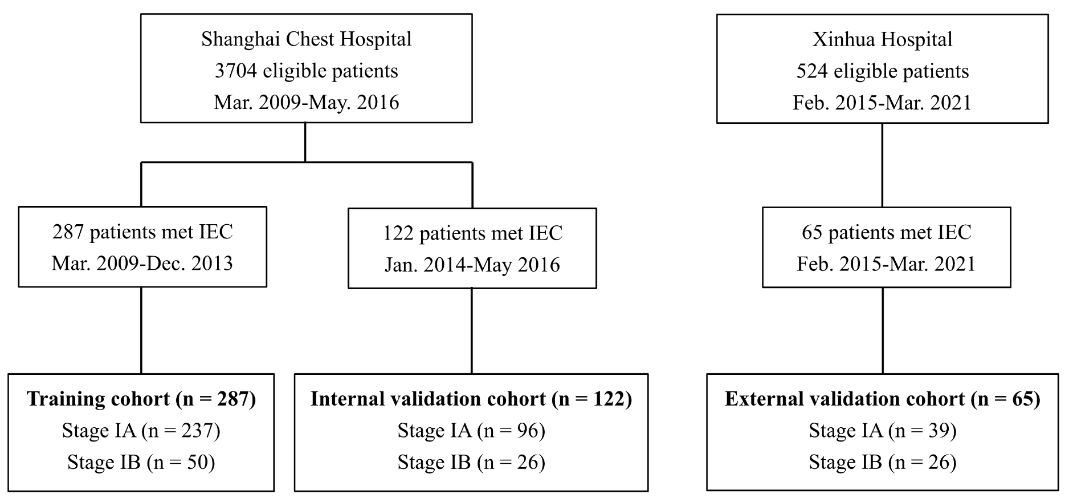


**Figure S1.** The process of patient selection in the training and two validation cohorts


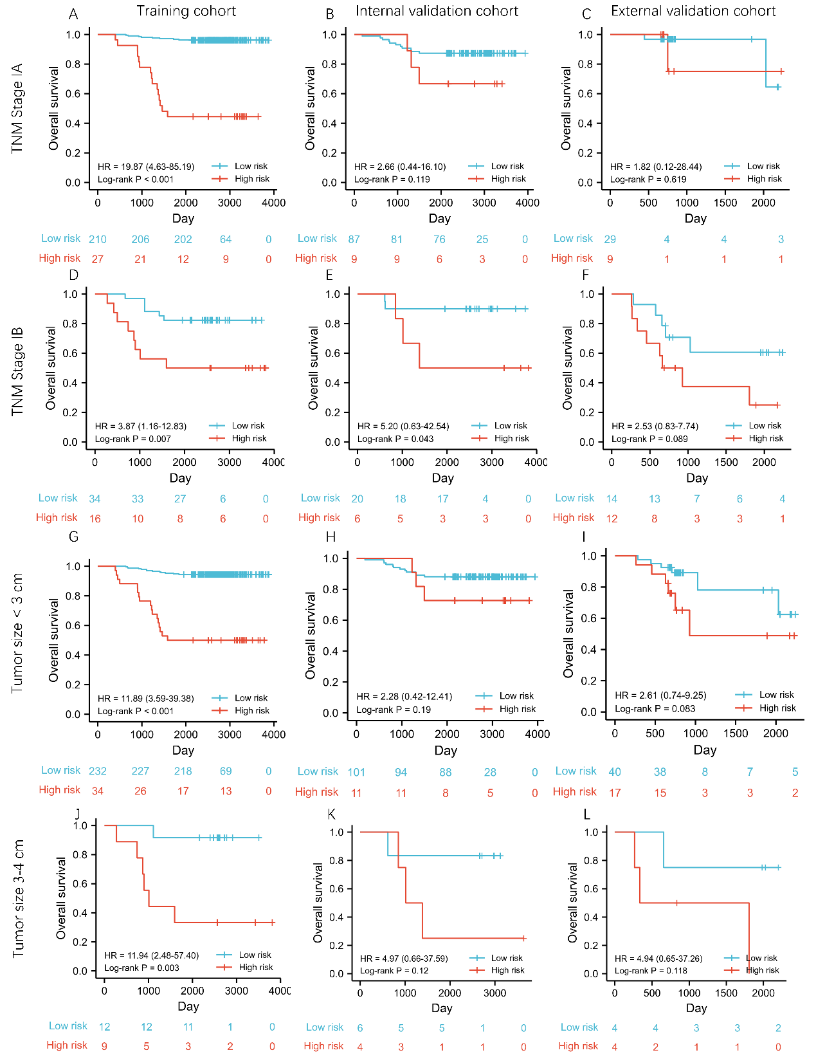


**Figure S2.** Kaplan-Meier overall survival curves according to the radiomic signature among stage I lung adenocarcinoma patient subgroups. The training cohort (A, D, G, J), The internal validation cohort (B, E, H, K), and external validation cohort (C, F, I, L). P values were calculated using two-sided log-rank test.


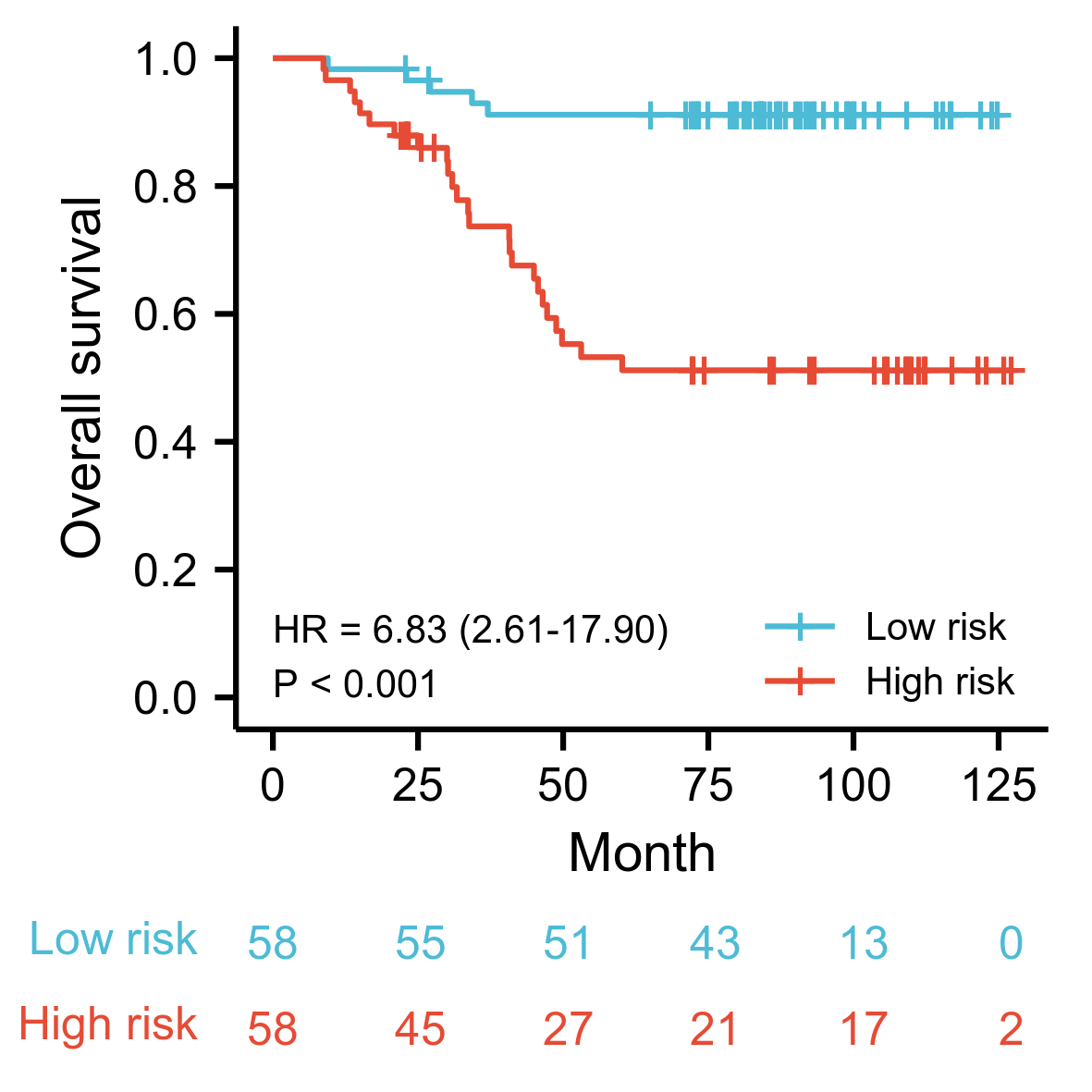


**Figure S3.** Kaplan-Meier overall survival curve according to the radiomic signature after propensity score matching.

**Table S1.** Multivariate Cox Regression analyses for overall survival in the training and validation cohorts

|  | HR (95% CI) | P value |
| --- | --- | --- |
| **Training cohort** |  |  |
| VPI (present vs. absent) | 2.348 (1.171-4.706) | 0.016 |
| RS (high vs. low risk) | 10.601 (5.279-21.291) | <0.001 |
| **Internal validation cohort** |  |  |
| Gender (female vs. male) | 0.234 (0.074-0.744) | 0.014 |
| Age | 1.086 (1.023-1.152) | 0.007 |
| VPI (present vs. absent) | 3.953 (1.342-11.643) | 0.013 |
| RS (high vs. low risk) | 2.628 (0.966-7.148) | 0.058 |
| **External validation cohort** |  |  |
| Age | 1.071 (0.997-1.151) | 0.062 |
| TNM stage (IB vs. IA) | 2.718 (1.051-7.026) | 0.039 |
| RS (high vs. low risk) | 3.879 (1.219-12.344) | 0.022 |

VPI, visceral pleural invasion; RS, radiomic signature; HR, hazard ratio; CI, confidence interval

**Table S2.** Demographic and clinicopathological characteristics of patients by radiomic signature level before and after propensity score matching.

|  | Before PSM | | | After PSM | | |
| --- | --- | --- | --- | --- | --- | --- |
|  | Low risk (n = 395) | High risk (n = 79) | P value | Low risk (n = 58) | High risk (n = 58) | P value |
| Age, median (IQR) | 60 (53-67) | 61 (53-67) | 0.987 | 61.5 (52-69.3) | 61.0 (53-67.3) | 0.969 |
| Gender (%) |  |  | 0.869 |  |  | 0.573 |
| Male | 179 (45.3) | 35 (44.3) |  | 26 (44.8) | 23 (39.7) |  |
| Female | 216 (54.7) | 44 (55.7) |  | 32 (55.2) | 35 (60.3) |  |
| Smoking history (%) |  |  | 0.132 |  |  | 0.590 |
| Never | 341 (86.3) | 63 (79.7) |  | 51 (87.9) | 49 (84.5) |  |
| Ever | 54 (13.7) | 16 (20.3) |  | 7 (12.1) | 9 (15.5) |  |
| Tumor location (%) |  |  | 0.035 |  |  | 0.126 |
| Upper left | 77 (19.5) | 19 (24.1) |  | 19 (32.8) | 12 (20.7) |  |
| Lower left | 52 (13.2) | 19 (24.1) |  | 5 (8.6) | 15 (25.9) |  |
| Upper right | 141 (35.7) | 27 (34.2) |  | 19 (32.8) | 18 (31.0) |  |
| Right middle | 40 (10.1) | 5 (6.3) |  | 4 (6.9) | 5 (8.6) |  |
| Lower right | 85 (21.5) | 9 (11.4) |  | 11 (19.0) | 8 (13.8) |  |
| Pathological subtype (%) |  |  | <0.001 |  |  | 0.304 |
| Lepidic | 56 (14.2) | 11 (13.9) |  | 8 (13.8) | 11 (19.0) |  |
| Acinar/papillary | 327 (82.8) | 55 (69.6) |  | 46 (79.3) | 39 (67.2) |  |
| Solid/micropapillary | 12 (3.0) | 13 (16.5) |  | 4 (6.9) | 8 (13.8) |  |
| Tumor size (cm) |  |  | <0.001 |  |  | 0.915 |
| 0-1 | 84 (21.3) | 8 (10.1) |  | 7 (12.1) | 8 (13.8) |  |
| 1-2 | 193 (48.9) | 23 (29.1) |  | 22 (37.9) | 20 (34.5) |  |
| 2-3 | 96 (24.3) | 31 (39.2) |  | 24 (41.4) | 23 (39.7) |  |
| 3-4 | 22 (5.6) | 17 (21.5) |  | 5 (8.6) | 7 (12.0) |  |
| TNM stage (%) |  |  | <0.001 |  |  | 0.691 |
| IA1 | 78 (19.7) | 9 (11.4) |  | 6 (10.3) | 9 (15.5) |  |
| IA2 | 168 (42.5) | 17 (21.5) |  | 18 (31.0) | 14 (24.1) |  |
| IA3 | 81 (20.5) | 19 (24.1) |  | 16 (27.6) | 14 (24.1) |  |
| IB | 68 (17.2) | 34 (43.0) |  | 18 (31.0) | 21 (36.2) |  |
| Visceral pleural invasion (VPI, %) |  |  | <0.001 |  |  | 0.837 |
| Absent | 344 (87.1) | 49 (62.0) |  | 41 (70.7) | 42 (72.4) |  |
| Present | 51 (12.9) | 30 (38.0) |  | 17 (29.3) | 16 (27.6) |  |
| Lymphovascular invasion (LVI, %) |  |  | 0.705 |  |  | 1.000 |
| Absent | 383 (97.0) | 78 (98.7) |  | 57 (98.3) | 57 (98.3) |  |
| Present | 12 (3.0) | 1 (1.3) |  | 1 (1.7) | 1 (1.7) |  |

PSM, propensity score matching; IQR, interquartile range

**Table S3**. The performances of the different models in the training and validation cohorts

| Cohort | C-index (95% CI) | | |
| --- | --- | --- | --- |
|  | Radiomic signature | Clinical nomogram | Radiomic nomogram |
| Training cohort | 0.838 (0.773-0.903) | 0.698 (0.616-0.780) | 0.852 (0.793-0.911) |
| Internal validation cohort | 0.719 (0.617-0.821) | 0.787 (0.691-0.883) | 0.838 (0.757-0.920) |
| External validation cohort | 0.712 (0.597-0.827) | 0.774 (0.696-0.852) | 0.863 (0.785-0.941) |

C-index, concordance index; CI, confidence interval
